# Medical consultations for the patients with severe mental illness: An evaluation in psychiatry inpatient service

**DOI:** 10.1192/j.eurpsy.2021.1731

**Published:** 2021-08-13

**Authors:** İ.G. Yılmaz Karaman, E. Ağrıdağ, F. Demircan

**Affiliations:** Psychiatry Department, Eskişehir Osmangazi University, Faculty of Medicine, Eskişehir, Turkey

**Keywords:** medical comorbidity, psychiatry inpatient service, Severe mental illness

## Abstract

**Introduction:**

Patients with severe mental illness die 10-20 younger from general population. In addition to suicide, preventable physical diseases cause most deaths. The mental illness itself and stigma keep the patients from adequate treatment for physical ilnesses.

**Objectives:**

We aimed to investigate medical consultations for inpatients with severe mental illnes.

**Methods:**

We retrospectively evaluated medical records of patients diagnosed by schizophrenia, schizoaffective disorders, bipolar disorder, and depression between 1st Februrary 2018 and 30th January 2020. We excluded routine consultations before electroconvulsive treatment. Local ethichs committee approved the study.

**Results:**

Among total 475 consultations, %41.3 (n=196) was for male, and %58.7 (n=279) was for female patients. Mean age and standart deviation were 48.9 ± 13.9 for male, and 50.1 ± 13.7 for female (p>0.05). Comparing sexes oin terms of primer psychiatric diagnoses, the higher proportion was psychotic disorders for male, and for female it was mood disorders (p<0.05). The most consulted departments with percentage and number were: internal medicine %44.0 (n=209), neurology and neurosurgery %15.2 (n=72), physical medicine and rehabilitation %8.2 (n=39), dermatology %7.8 (n=37), cardiology %6.7 (n=32). We compared the proportions of consulted department between male and female. Male patients were consulted to dermatology more than female, and female patient were consulted to gynecology or urology more than male (p<0.05).

**Conclusions:**

Awareness about physical diseases in paients with severe mental illness between healthcare workers, carries the potential to increase the patients’ quality of life and lifespan. For future interventions the focus should involve healthcare worker in internal medicine and neurology, as well as in psychiatry.

**Disclosure:**

No significant relationships.

